# The metabolic profile of reconstituting T-cells, NK-cells, and monocytes following autologous stem cell transplantation and its impact on outcome

**DOI:** 10.1038/s41598-022-15136-3

**Published:** 2022-07-06

**Authors:** Silja Richter, Martin Böttcher, Simon Völkl, Andreas Mackensen, Evelyn Ullrich, Benedikt Jacobs, Dimitrios Mougiakakos

**Affiliations:** 1 Department of Internal Medicine 5, Hematology and Clinical Oncology, Friedrich-Alexander-Universität Erlangen-Nürnberg (FAU), University Hospital Erlangen, Erlangen, Germany; 2 Department of Hematology, Oncology, and Stem Cell Transplantation, Otto-Von-Guericke-University Magdeburg, University Hospital, Leipziger Strasse 44, 39120 Magdeburg, Germany; 3 Deutsches Zentrum Für Immuntherapie, Friedrich-Alexander-Universität Erlangen-Nürnberg (FAU), Erlangen, Germany; 4 Children’s Hospital, Goethe-University Frankfurt, Frankfurt, Germany; 5 Experimental Immunology, Goethe University Frankfurt, Frankfurt, Germany; 6 Frankfurt Cancer Institute, Goethe University Frankfurt, Frankfurt, Germany

**Keywords:** Immunology, Tumour immunology, Lymphoma, Myeloma

## Abstract

Previous studies indicated a role of the reconstituting immune system for disease outcome upon high-dose chemotherapy (HDCT) and autologous stem cell transplantation (auto-SCT) in multiple myeloma (MM) and lymphoma patients. Since immune cell metabolism and function are closely interconnected, we used flow-cytometry techniques to analyze key components and functions of the metabolic machinery in reconstituting immune cells upon HDCT/auto-SCT. We observed increased proliferative activity and an upregulation of the glycolytic and fatty acid oxidation (FAO) machinery in immune cells during engraftment. Metabolic activation was more pronounced in T-cells of advanced differentiation stages, in CD56^bright^ NK-cells, and CD14^++^CD16^+^ intermediate monocytes. Next, we investigated a potential correlation between the immune cells’ metabolic profile and early progression or relapse in lymphoma patients within the first twelve months following auto-SCT. Here, persistently increased metabolic parameters correlated with a rather poor disease course. Taken together, reconstituting immune cells display an upregulated bioenergetic machinery following auto-SCT. Interestingly, a persistently enhanced metabolic immune cell phenotype correlated with reduced PFS. However, it remains to be elucidated, if the clinical data can be confirmed within a larger set of patients and if residual malignant cells not detected by conventional means possibly caused the metabolic activation.

## Introduction

HDCT followed by auto-SCT is a well-established treatment option for different hematological malignancies including MM, Hodgkin (HL) and non-Hodgkin lymphomas (NHL). For newly diagnosed MM (NDMM) induction therapy followed by HDCT/auto-SCT remains the state of the art first-line treatment option for transplantation-eligible patients despite the introduction of various novel agents^1^. For mantle cell (MCL) and peripheral T-cell lymphoma (TCL) patients the use of HDCT/auto-SCT as a consolidation treatment after induction chemotherapy increases progression-free survival (PFS) and overall survival (OS)^2–4^. Similar results have been observed for primary central nerve system lymphoma (PCNSL)^5^. In addition, about 50% of refractory or relapsed (r/r) diffuse-large B cell lymphoma (DLBCL) patients who respond to salvage treatment and proceed to HDCT/auto-SCT will achieve a sustained remission. Unfortunately, the other half will ultimately relapse and will have a poor prognosis^6,7^. Identifying risk factors that predict relapse or refractoriness upon auto-SCT has been the focus of several studies assessing amongst others the disease status at transplantation, tumor burden or the time to the (preceding) relapse^8,9^. Moreover, the patients’ immune system appears to be a determinant of disease outcome following auto-SCT. A timely and stable lymphocyte reconstitution is associated with improved PFS and in part OS in various malignancies including DLBCL, MM, MCL, TCL but also solid tumors like breast and ovarian cancer^10^.

Function, proliferation, and survival of immune cells critically depend on their metabolic fitness^11^. In general, dormant cells preferentially meet their energetic demands through mitochondrial oxidative phosphorylation (OXPHOS)^12^. In contrast, proliferating (and/or activated) cells particularly utilize aerobic glycolysis that is less effective leading to fewer ATP per consumed glucose molecule. This phenomenon was first described by Otto Warburg in cancer cells and is known as the “Warburg Effect”^13,14^. In fact, increasing evidence suggest that immune metabolic competence is a prerequisite for a proper anti-tumor immune response and is regularly hampered by tumors^15^. T-cells from CLL patients display reduced expression of glucose transporters and an overall suppressed glucose metabolism that is driving their functional impairment^16^. NK-cells within the lymphoma microenvironment (including DLBCL) upregulate their lipid metabolism^17^ resulting in attenuated effector functions. PD-L1 expressed on CLL-cells triggers PD-1 on circulating monocytes, which silences their metabolic activity thereby leading amongst others to reduced tumoricidality^18^. In fact, metabolic reprogramming of reconstituting donor T-cells following allogeneic stem cell transplantation (allo-SCT) was recently shown to impact their ability to control residual acute myeloid leukemia (AML) blasts and thus to prevent relapse^19^.

As of to date, no data on the metabolic phenotype of reconstituting T-cells, NK-cells, and monocytes following auto-SCT is available. Here, we provide such an assessment in patients with MM and lymphoma that underwent an auto-SCT and correlate our findings with the clinical outcome.

## Results

### Absolute T- and NK-cell but not monocyte count is significantly reduced at engraftment time following HDCT/auto-SCT

**Figure 1:**
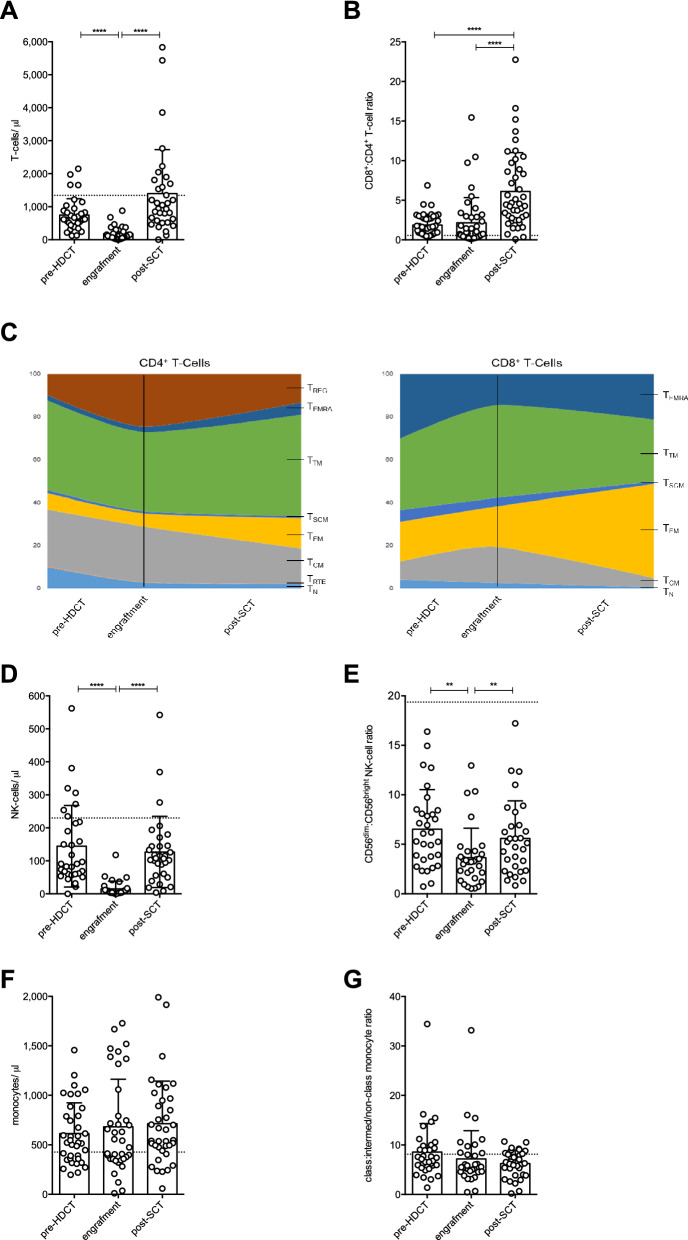
Absolute T- and NK-cell but not monocyte count is significantly reduced at engraftment time following HDCT/ auto-SCT. (**A**) Analysis of absolute CD3^+^ T-cell count in MM and lymphoma patients (n: 34; mean +/− standard deviation SD) during HDCT/ auto-SCT. (**B**) Course of CD8^+^:CD4^+^ T-cell ratio (n: 43; mean+/− SD). (**C**) Distribution of various differentiation stages of CD4^+^ and CD8^+^ T-cells (n: 10) including naïve (N), recent thymic emigrants (RTE), stem cell memory (SCM), central memory (CM), transitional memory (TM), effector memory (EM), effector memory CD45RA^+^ (EMRA) and regulatory T-cells (REG)*.* (**D**) Analysis of absolute NK-cell count (n: 32; mean +/− SD) during HDCT/ auto-SCT. (**E**) Course of CD56^dim^:CD56^bright^ NK-cell ratio (n: 31; mean +/− SD). (**F**) Analysis of absolute monocyte count (n: 37; mean +/− SD) during HDCT/ auto-SCT. (**G**) Course of classical:intermediate/non-classical monocyte ratio (n: 34; mean +/− SD). The mean of a healthy donor control group (n: 52 from A.H. Kverneland et al.^61^) is shown as a horizontal dashed line for reference. Statistical significance was determined using a Friedman test with Dunn’s multiple comparison tests for repeated measurements. **P < .01; ****P < .0001.

Several studies in different malignancies indicated that a stable lymphocyte reconstitution upon auto-SCT and a lymphocyte to monocytes ratio ≥ 1 are both associated with improved PFS and OS^10^. Therefore, we monitored the lymphocyte and monocyte count within our patient population, thereby excluding B-cells since they were usually not detected in lymphoma patients due to prior rituximab therapy. The number of CD3^+^ T-cells was significantly reduced at the time of engraftment (746 vs. 180 per µl; p < 0.0001) and fully recovered at the restaging appointment (Fig. 1A). Interestingly, we noticed a skewing towards CD8^+^ T-cells over the course of hematopoietic reconstitution (Fig. 1B). Next, we investigated the dynamics of T-cell differentiation including naturally occurring regulatory (T_REG_), naïve (T_N_), recent thymic emigrant (T_RTE_), stem cell memory (T_SCM_), central memory (T_CM_), transitional memory (T_TM_), effector memory (T_EM_), and effector memory re-expressing CD45RA (T_EMRA_) T-cells (Supplementary Table S1 and Figure S1 online)^20,21^. Within the CD4^+^ T-cell compartment, early differentiation stages (i.e., T_N_, T_SCM_, and T_RTE_) decreased. In contrast, proportion of all memory phenotypes (except T_CM_) and of T_REG_ increased to higher levels as compared to the pre-HDCT situation. Among CD8^+^ T-cells we observed a decrease of T_N_ and T_SCM_ cells. At engraftment T_CM_ and T_TM_ frequencies increased and T_EMRA_ decreased while T_EM_ remained stable, which was followed by a contraction of T_CM_, and T_T__M_, and a remarkable expansion of T_EM_ (Fig. 1C and Supplementary Table S2 online).

Kinetics of circulating NK-cells was equivalent to the one of T-cells (Fig. 1D). NK-cells are divided into the CD56^bright^ (CD56^++^CD16^-^) and CD56^dim^ (CD56^+^CD16^+/−^) subset (Supplementary Figure S1 online)^22^. The ratio of CD56^bright^:CD56^dim^ NK-cells shifted to CD56^bright^ NK-cells to return to prior-HDCT levels (Fig. 1E).

In contrast to T- and NK-cells, monocyte numbers remained stable following HDCT/auto-SCT (Fig. 1F). Monocytes can be subdivided into a classical (CD14^++^CD16^-^), intermediate (CD14^++^CD16^+^), and non-classical (CD14^dim^CD16^++^) subset (Supplementary Figure S1 online). HDCT/auto-SCT did not impact the composition of the monocyte population (Fig. 1G).

Since the duration from auto-SCT until the restaging appointment significantly differed between myeloma and lymphoma patients (Table 1), we further compared absolute cell numbers between the two patients groups at this time point. However, no significant differences were observed for neither of the three cell subsets (Supplementary Figure S2 online).

**Table 1:** Characteristics of HDCT/ auto-SCT patients.

	All	Myeloma	Lymphoma
**Number of patients**	53	30	23
DLBCL			13
MCL			2
MZoL			1
FL			1
HD			1
TCL			5
**Gender**			
Male	35	18	17
Female	18	12	6
**Age, median (range)**	60 (32–76)	60.5 (42–72)	59 (32–76)
**No. of previous therapies, median (range)**	1 (1–3)	1 (1–3)**	2 (1–3)**
**Time from last chemo to start of HDCT (days), median (range)**	31 (17–70)	41 (24–70)****	25 (17–42)****
**Time from SCT to engraftment (days), median (range)**	11 (9–19)	11 (9–19)	11 (9–14)
**Time from SCT to re-staging (days), median (range)**	39 (23–66)	45 (28–66)****	34 (23–57)****
**CD34**^**+**^** cell number (×10**^**6**^**/kg), median (range)**	4.14 (2.6–17.6)	4.0 (2.74–8.1)	4.14 (2.6–17.6)
**Treatment regime HDCT**			
Melphalan		30	
(R)/O-BEAM			16
(R)-BCNU-TT			6
R-BendaEAM			1

DLBCL, diffuse large B-cell lymphoma; MCL, mantle cell lymphoma; MZol, marginal zone lymphoma; FL, follicular lymphoma; HD, Hodgkin disease; TCL, T-cell lymphoma; HDCT, high-dose chemotherapy, R-/O-BEAM, rituximab/ obinutuzumab, carmustine, etoposide, cytarabine, melphalan; R-BCNU-TT: rituximab, carmustine, thiothepa; R-BendaEAM, rituximab, bendamustin, etoposide, cytarabine, melphalan.

Statistical significance was determined using a Mann–Whitney test for unpaired, non-parametric variables and a Chi-square test for categorical variables. P values comparing myeloma and lymphoma patients **P < .01, ****P < .0001.

### T-cells, NK-cells, and monocytes maintain their ability to produce cytokines following HDCT/auto-SCT

**Figure 2:**
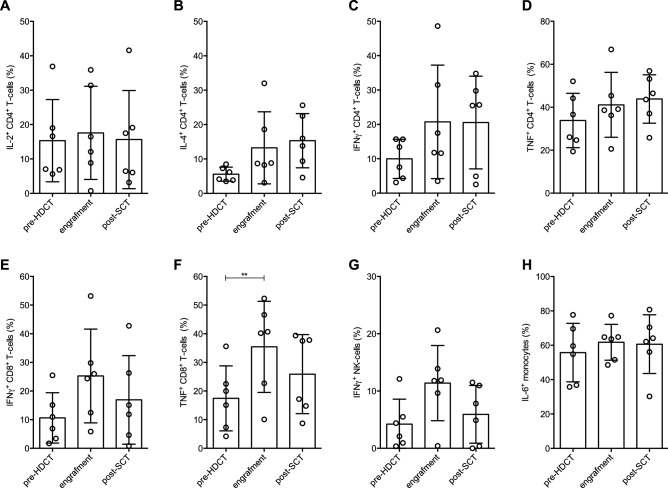
T-cells, NK-cells, and monocytes maintain their ability to produce cytokines following HDCT/auto-SCT. (**A**–**D**) Illustration of the percentages of IL-2 (**A**), IL-4 (**B**), IFN-γ (**C**) and TNF (**D**) positive CD4^+^ T-cells during HDCT/ auto-SCT upon stimulation with Phorbol-12-myristat-13-acetat (PMA)/ Ionomycin. (**E–G**) Plotted are the percentages of IFN-γ (**E**) and TNF (**F**) positive CD8^+^ T-cells as well as of IFN-γ positive NK-cells (**G**) at the indicated time points. (**H**) Percentage of IL-6 positive monocytes upon LPS stimulation (n: 6, mean values +/− SD). Statistical significance was determined using a Friedman test with Dunn’s multiple comparison tests for repeated measurements. **P < .01.

Next, we were interested whether reconstituting immune cells were capable of cytokine production. In CD4^+^ T-cells IL-2 production was stable, whereas IL-4, IFN-γ, and TNF displayed elevated levels. Which was similarly observed for IFN-γ and TNF in their CD8^+^ counterparts. However, only TNF production in CD8^+^ T-cells reached statistical significance, probably due to the low number of tested patients (Fig. 2A–F). Similarly, pro-inflammatory cytokine production by NK-cells (i.e., IFN-γ) and monocytes (i.e., IL-6) was not negatively affected. Rather, production of IFN-γ by NK-cells was highest at the time of engraftment (Fig. 2G,H).

### T-cells, NK-cells, and monocytes display increased proliferative activity and an enhanced glycolytic machinery during reconstitution upon HDCT/auto-SCT

**Figure 3:**
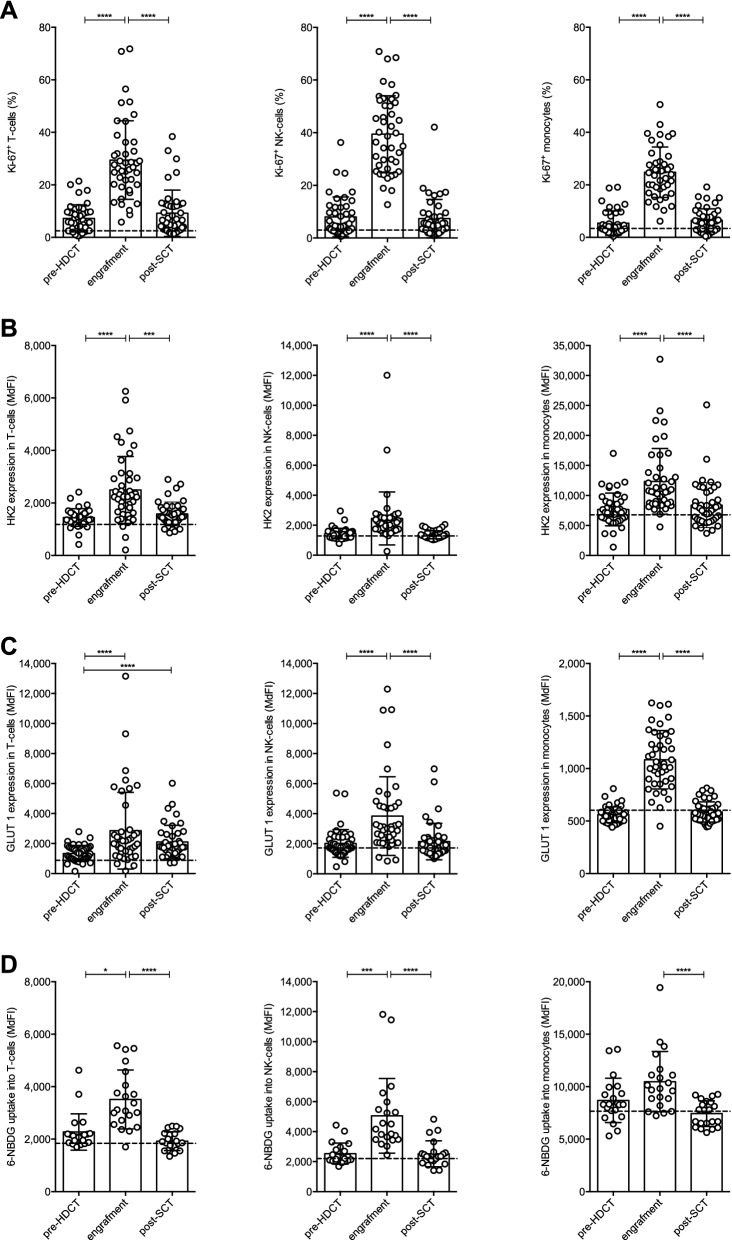
T-cells, NK-cells, and monocytes display increased proliferative activity and an enhanced glycolytic machinery during reconstitution upon HDCT/auto-SCT. (**A**) Proliferation is illustrated by plotting the percentages of Ki-67^+^ cells during all three time points. (**B**) The expression of the rate-limiting enzyme of glycolysis hexokinase 2 (HK2) was measured. (**C**) The abundance of the glucose transporter GLUT1 was evaluated. (**D**) To measure the cells’ glucose uptake the fluorescent glucose analogue 6-NDBG was used and plotted as median fluorescence intensity (MdFI) for all cell populations (n: 43, for Ki-67, HK2 and GLUT1, n: 21 for 6-NBDG; mean values +/− SD). The mean of a healthy donor control group (n: 20 for Ki-67, HK2 and GLUT1, n: 10 for 6-NBDG) is shown as a horizontal dashed line for reference. Statistical significance was determined using a Friedman test with Dunn’s multiple comparison tests for repeated measurements. *P < .05; ***P < .001; ****P < .0001.

Early and robust immune cell reconstitution upon auto-SCT is associated with less infectious complications and improved tumor surveillance^10^. During engraftment T-cells, NK-cells, and monocytes were highly proliferative as indicated by the high frequency of Ki-67^+^ cells (Fig. 3A). It is well established, that proliferating immune cells maintain high levels of (aerobic) glycolysis^11^. As anticipated, we found in all investigated types of cells an increased expression of hexokinase 2 (HK2), the rate-limiting enzyme of glycolysis (Fig. 3B). This was accompanied by an abundance of glucose transporter 1 (GLUT1, Fig. 3C) and an increased glucose uptake (Fig. 3D). Over time, all glycolytic parameters examined normalized together with the cells’ proliferative activity (Fig. 3A–D).

### FAO and mitochondrial parameters are increased in T-cells, NK-cells, and monocytes upon HDCT/auto-SCT

**Figure 4:**
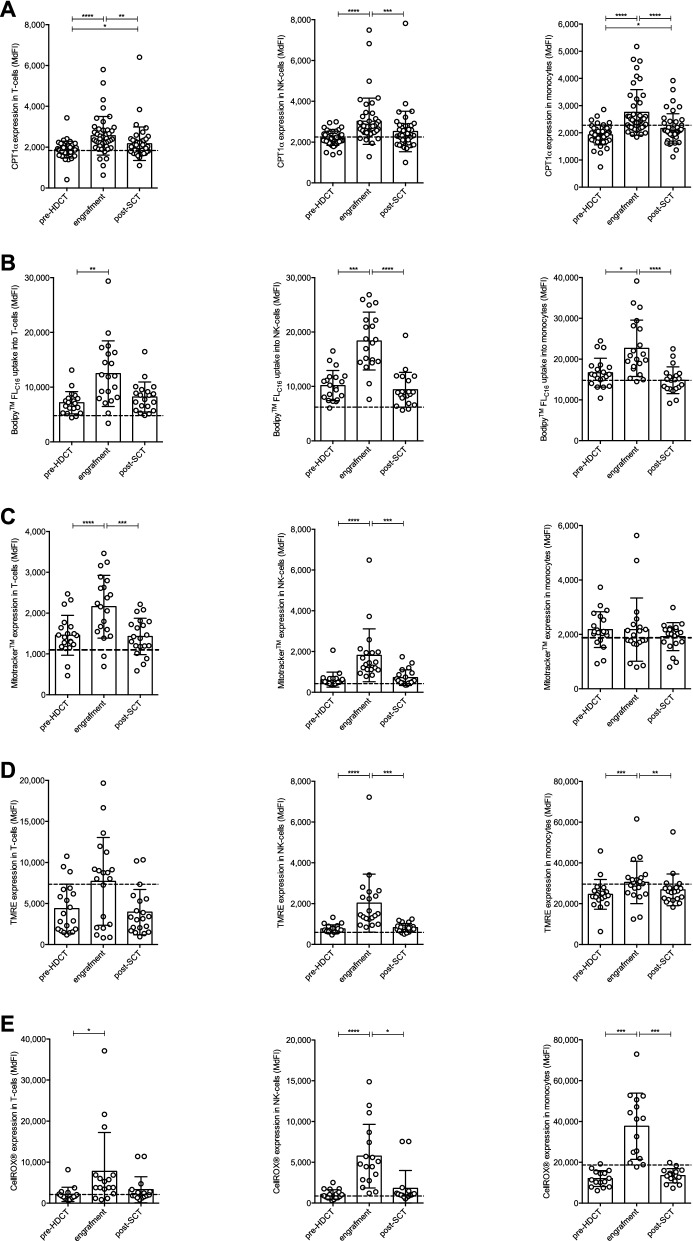
FAO and mitochondrial parameters are increased in T-cells, NK-cells, and monocytes upon HDCT/auto-SCT. (**A**) The FAO pathway was analyzed by measuring the expression of CPT1α. (**B**) Uptake of fatty acids was measured using the fluorescent fatty acid analogue Bodipy FL_C16_. (**C**) Mitochondrial mass was measured using Mitotracker. (**D**) Mitochondrial membrane potential was measured using TMRE. (**E**) Cellular ROS production was evaluated using CellROX Deep Red reagent. All results were plotted as median fluorescence intensity (MdFI) for all cell populations (n: 15–43, mean values +/− SD). The mean of a healthy donor control group (n: 10–19) is shown as a horizontal dashed line for reference. Statistical significance was determined using a Friedman test with Dunn’s multiple comparison tests for repeated measurements. *P < .05; **P < .01; ***P < .001; ****P < .0001.

Beyond glycolysis, FAO and mitochondrial metabolism are essential elements of the immune cells’ homeostasis and function^23^. In analogy to HK2, carnitine palmitoyl-transferase I alpha (CPT1α), the rate-limiting enzyme of the FAO pathway, was significantly increased during engraftment in all three cell populations. Of note, while CPT1α levels decreased back to baseline levels in NK-cells, they remained elevated in T-cells and monocytes (Fig. 4A). Increased CPT1α expression during engraftment was accompanied by elevated uptake of fluorescent fatty acid analogues (Bodipy FL_C16_) (Fig. 4B). Mitochondria represent central metabolic hubs. Mitochondrial mass was increased in T- and NK-cells but not monocytes during engraftment and returned to baseline levels at later time points (Fig. 4C). Mitochondrial potential as a surrogate for mitochondrial fitness and an indicator for mitochondrial activity was elevated in all cell types during engraftment (Fig. 4D). There are several sources for reactive oxygen species (ROS) in a cell. Mitochondria are a major site for ROS production, which takes place at the electron transport chain during OXPHOS. We found elevated global ROS levels in all cell types during engraftment, which is consistent with their increased metabolic activity (Fig. 4E). However, mitochondrial ROS production was only significantly increased in NK-cells during engraftment, but not in T-cells or monocytes (Supplementary Figure S3 online).

### Proliferative activity and metabolic armamentarium are not homogenous within T-cell, NK-cell, and monocyte subpopulations following HDCT/auto-SCT

**Figure 5:**
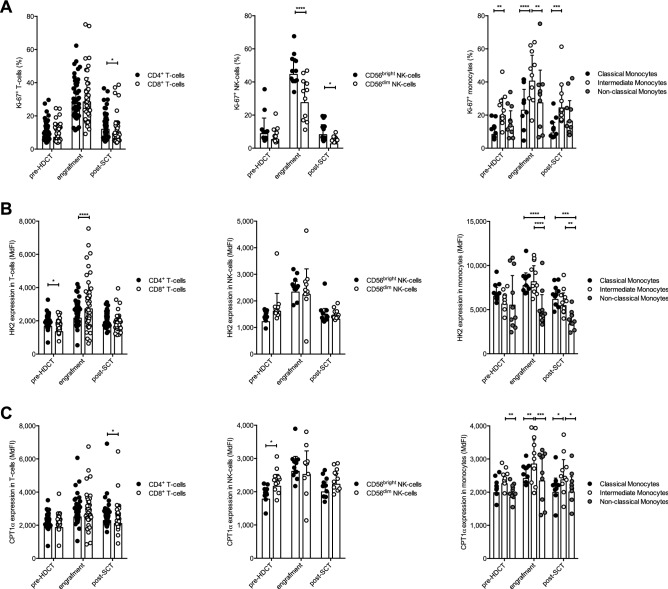
Proliferative activity and metabolic armamentarium are not homogenous within T-cell, NK-cell, and monocyte subpopulations following HDCT/auto-SCT. (**A**) Proliferation of T- and NK-cell as well as monocyte subsets was evaluated by plotting the percentages of Ki-67^+^ cells during the three different time points. (**B**) The glycolytic pathway was analyzed by measuring the expression of HK2. (**C**) The FAO pathway was analyzed by measuring the expression of CPT1α. All results of the latter two markers were plotted as median fluorescence intensity (MdFI) for all cell populations (n: 43 (T-cells), 11 (NK-cells), 10 (monocytes), mean values +/− SD). Statistical significance was determined using a two-way ANOVA with Sidak’s multiple comparison tests for paired comparisons between T-, NK-cell and monocyte subsets. *P < .05; **P < .01; *** < .001; ****P < .0001.

Next, we assessed, whether the observed alterations in terms of proliferation and expression of glycolysis or FAO pacemaker enzymes following HDCT/auto-SCT are consistent among the major subsets of T-cells, NK-cells, and monocytes. Proliferation was increased at engraftment compared to pre-HDCT in all investigated major subsets. The proliferating fraction of CD4^+^ and CD8^+^ T-cells was comparable at engraftment. CD56^bright^ NK-cells displayed a superior proliferative activity as compared to their CD56^dim^ counterpart. Finally, fraction of proliferative cells was highest amongst intermediate monocytes at all investigated time points (Fig. 5A). In terms of HK2, reconstituting CD8^+^ T-cells had significantly higher levels as compared to CD4^+^ T-cells. No differences could be observed for CD56^bright^ and CD56^dim^ NK-cells. In addition, classical and intermediate monocytes demonstrated significantly higher HK2 levels during engraftment and restaging as compared to non-classical ones, which did not demonstrate elevated HK2 levels at restaging as compared to the pre-HDCT timepoint (Fig. 5B). CPT1α levels increased upon auto-SCT and were similar in the analyzed T- and NK-cell compartments during engraftment. In fact, CPT1α content was higher in reconstituted CD4^+^ T-cells and lower in CD56^dim^ NK-cells prior to HDCT. Intermediate monocytes displayed the highest CPT1α expression during all investigated time points (Fig. 5C).

Taken together, proliferative activity and expression of metabolic pacemaker molecules display a heterogeneous pattern amongst T-cell, NK-cell, and monocyte subsets following auto-SCT, with proliferation not always positively correlating with HK2 and/or CPT1α expression (as an expression of an increased energetic demand).

### CD4^+^ and CD8^+^ T-cells at early differentiation stages were less proliferative and less metabolically active

**Figure 6:**
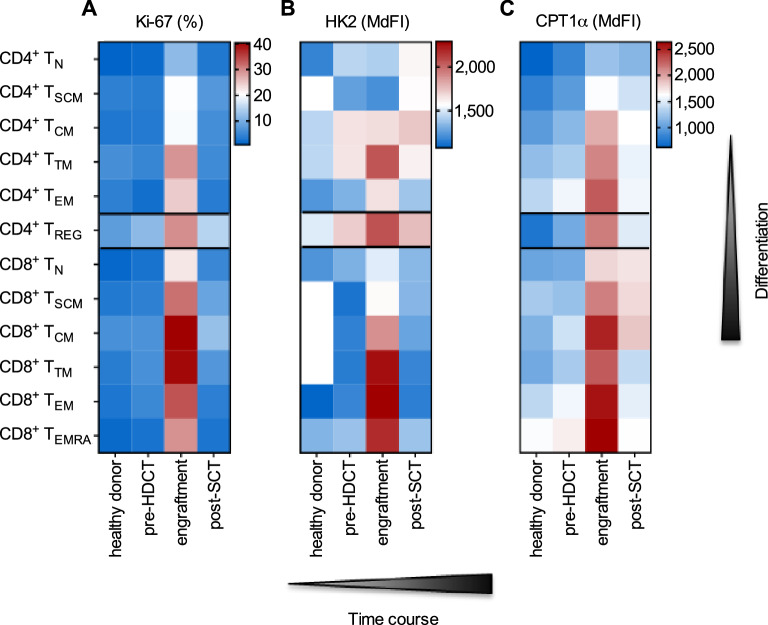
CD4^+^ and CD8^+^ T-cells at early differentiation stages were less proliferative and less metabolically active. (**A**) Proliferation of CD4^+^ and CD8^+^ T-cell differentiation stages including naïve (N), stem cell memory (SCM), central memory (CM), transitional memory (TM), effector memory (EM), effector memory CD45RA^+^ (EMRA) and regulatory T-cells (REG) was evaluated by color-coding the percentages of Ki-67^+^ cells. (**B**) The glycolytic pathway was analyzed by measuring HK2 expression. (**C**) The FAO pathway was analyzed by measuring CPT1α expression. All results of the latter two markers were color-coded based on the median fluorescence intensity (MdFI) for all cell populations (n: 10; mean values). The mean of a healthy donor control group (n: 10) was included for reference.

After characterizing the two major T-cell subsets, we aimed to extend our assessment to the area of T-cell differentiation including T_REG_, T_N_, T_SCM_, T_TM_, T_EM_, T_EMRA_, and T_CM_ cells (Supplementary Figure S1 online)^21^. In fact, during engraftment early differentiation stages such as T_N_ and T_SCM_ cells had low proliferation rates (Fig. 6A) as well as low HK2 (Fig. 6B) and CPT1α (Fig. 6C) expression levels as compared to later differentiation stages (i.e., T_CM_, T_TM_ and T_EM_ CD4^+^/CD8^+^ cells). CD4^+^ and CD8^+^ T_TM_ were strongly proliferative and were HK2^high^ during engraftment. High CPT1α levels were observed within CD4^+^ and CD8^+^ T_EM_ as well as within CD8^+^ T_CM_ cells at this time point. In addition, CD4^+^ T_REG_ cells were (like CD4^+^ T_TM_ cells) Ki-67^high^, HK2^high^, and CPT1α^high^. Despite that CD8^+^ T_EMRA_ cells did not show a strong proliferation rate as compared to CD8^+^ T_TM_ and T_CM_ cells, they demonstrated a HK2^high^ and CPT1α^high^ phenotype (Fig. 6).

In summary, our data indicates that early differentiation stages of both CD4^+^ and CD8^+^ conventional T-cells are less metabolically active, whereas CD4^+^ T_REG_ cells have an activated metabolic profile during engraftment.

### Persistently increased metabolic activity upon HDCT/auto-SCT is associated with refractory disease or early relapse in lymphoma

**Figure 7:**
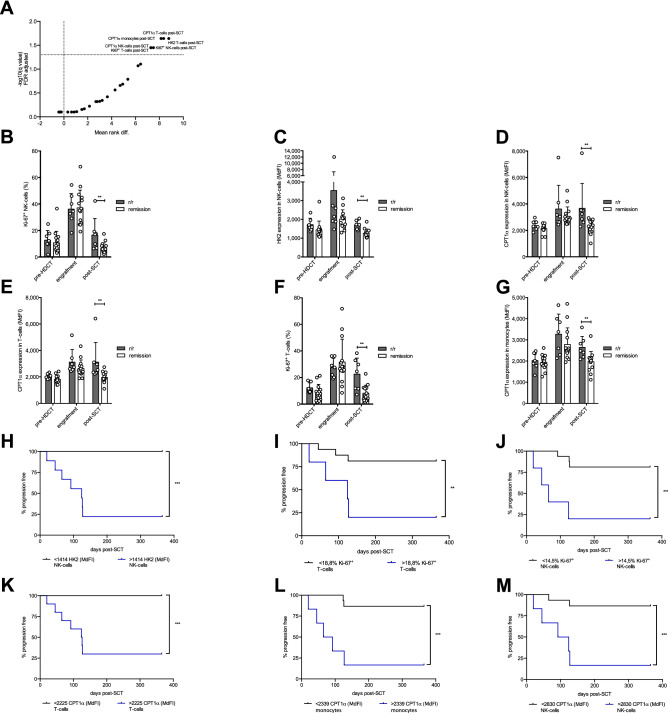
Persistently increased metabolic activity upon HDCT/ auto-SCT is associated with refractory or early relapse in lymphoma. (**A**) Multiple unpaired Mann–Whitney tests for Ki-67, HK2 and CPT1α expression in T-, NK-cells and monocytes at all three time points were performed to identify significant differences between lymphoma patients being refractory or relapsed (r/r) within the first year upon SCT and lymphoma patients being still in remission (remission) at this time point. P-values were adjusted using False-Discovery-Rate adjustment. (**B**) Ki-67, (**C**) HK2 and (**D**) CPT1α expression in NK-cells as well as (**E**) CPT1α and (**F**) Ki-67 in T-cells and (**G**) CPT1α expression in monocytes were plotted. All results of the CPT1α and HK2 expression are plotted as median fluorescence intensity (MdFI) whereas Ki-67 is plotted as percentages of all Ki-67^+^ cells (n: 7 refractory/ relapsing and 14 non-relapsing lymphoma patients, mean values +/− SD). (**H-M**) Area under the Receiver operating characteristic (ROC) curve analysis was used to define an optimal cutoff value for each parameter for further evaluation and grouping for survival analysis (Supplementary Figure S3–8). Cut off values were selected according to highest sensitivity (true positive rate) and specificity (true negative rate) and high Likelihood ratio (LR). Tumor progress within one year was recorded as an event. For comparison of progression free survival curves a Log-rank (Mantel Cox) test was performed. *P < .05; **P < .01; ***P < .001.

Finally, we were interested in whether the metabolic profile of T-cells, NK-cells or monocytes can be correlated with the clinical outcome in lymphoma patients^8–10^. Therefore, we compared the cells’ Ki-67, HK2, and CPT1α expression levels in patients in remission *versus* patients with refractory disease or early relapse during the first year upon HDCT/auto-SCT. By applying multiple unpaired Mann–Whitney tests and False-Discovery-Rate adjustments we identified HK2 for NK-cells, Ki-67 for T- and NK-cells, and CPT1α for all three analyzed cell types at the time point of the restaging (i.e., post-SCT) as discriminators for both patient groups (Fig. 7A). Interestingly, NK-cells displayed upregulated Ki-67, HK2, and CPT1α levels during engraftment that remained elevated in the patient group with r/r lymphoma (Fig. 7B–D). Similar patterns were also noticed for CPT1α and Ki-67 in T-cells and for CPT1α in monocytes respectively (Fig. 7E–G).

To obtain the optimal cut-off values of the continuous variables Ki-67, HK2, and CPT1α that correlates with PFS, area under the ROC curve analyses were performed (Supplementary Figure S4–9 online). In fact, we were able to identify expression (cut-off) levels for HK2 in NK-cells, for Ki-67 in T- and NK-cells, and for CPT1α in T-cells, NK-cells, and monocytes significantly correlating with PFS (Fig. 7H–M). Since we observed a significant skewing of the CD8^+^:CD4^+^ T-cell ratio during reconstitution, we separately analyzed the impact of the metabolic profile of CD8^+^ and CD4^+^ T-cells on the clinical outcome of lymphoma patients. We identified Ki-67 for CD8^+^ T-cells, and CPT1α for CD4^+^ and CD8^+^ T-cells to be significantly different between the two patient groups during restaging (Supplementary Figure S10 online). Again we were able to identify expression (cut-off) levels for the three markers significantly correlating with PFS (Supplementary Figure S11–13 online).

To exclude confounding clinical parameters, we compared time from last therapy to HDCT and from auto-SCT to engraftment and restaging in the remission *versus* the r/r group and could not detect any differences. Moreover, no significant differences were observed concerning gender distribution, age, number of previous therapies or the number of infused CD34^+^ hematopoietic stem cells. Finally, although it has been previously reported that lymphoma patients with lower absolute lymphocyte counts (ALC) upon auto-SCT had an inferior PFS and OS^10^, we observed no difference between the two groups. In addition, the time of relapse from auto-SCT ranged from several days before restaging until 99 days afterwards in our patient cohort, with at least two patients who have already relapsed during the last analysis. The clinical data is summarized in Supplementary Table S3 online.

## Discussion

HDCT/auto-SCT can induce long-term remission in several hematological malignancies. A rapid reconstitution of immune cells including lymphocytes and NK-cells is associated with an improved PFS and overall survival (OS)^10,24–26^. Mounting evidence suggests an interrelation of immune cell proliferation, function, and metabolism. In fact, following HDCT/auto-SCT we observed an upregulation of the glycolytic key enzyme HK2 and of glucose transporters as well as an enhanced glucose uptake in T-cells, NK-cells, and monocytes. CPT1α that is critical for FAO was also found increased in all tested immune cell types. Furthermore, T- and NK-cells demonstrated enhanced mitochondrial biogenesis during engraftment. These changes of the metabolic phenotype were accompanied by an increased proliferative activity and a superior production of pro-inflammatory cytokines by CD8^+^ T-cells.

T-cells undergo metabolic reprograming upon activation to be able to rapidly proliferate and to carry out their effector functions. They activate glycolysis to rapidly generate bioenergetic substrates and biosynthetic precursors, which is in line with our observations^27^. We also observed increased CPT1α expression during engraftment, a feature that is typically seen in memory T-cells^27^. Fittingly, frequency of memory T-cells was increased at this time point. Moreover, mitochondrial biogenesis was enhanced as well, which has been observed in memory and effector T-cells^28^. In addition, IL-15 levels are elevated at engraftment, which triggers the metabolic master regulator mammalian target of rapamycin (mTOR) leading amongst others to enhanced glycolysis, FAO, and mitochondrial biogenesis^29–31^.

Furthermore, robust IFN-γ production by NK-cells early after auto-SCT is in line with our previous observations that NK-cells are active early upon auto-SCT with preserved degranulation and production of cytokines and chemokines in response to target cell encounter^32^. The link between cytokine-induced metabolic reprogramming of NK-cells and functionality has been well described. IFN-γ production by CD56^bright^ NK-cells upon cytokine stimulation depends on both glycolysis and oxidative phosphorylation^33^. Blocking glycolysis abolishes the NK-cells’ ability to control CMV infections, while treatment with the IL-15 super-agonist ALT-803 promotes CMV clearance and even prevents CMV reactivation upon allogeneic SCT^34^. However, the exact role of FAO in NK-cells is not unambiguously clarified. Increased lipid uptake can inhibit degranulation and IFN-γ production in NK-cells and blocking fatty acid transport into the mitochondria can restore NK-cell cytotoxicity^35^. Contrariwise, it has been demonstrated that NK-cells treated intermittently with IL-15 possess a superior capacity for degranulation and IFN-γ production together with high levels of CPT1α expression^36^.

In line with previous reports, we observed a significant drop of the T- and NK-cell count during engraftment, which normalized over time^37^. Moreover, it has been shown that CD4^+^ T-cell recovery is delayed, which is partly explained by a reduced number of naïve precoursors^37,38^. Accordingly, we document a reduced CD4 to CD8 ratio, which is paralleled by reduced early differentiation stages. T-cell reconstitution upon auto-SCT is achieved by mainly two mechanisms: homeostatic proliferation and thymopoiesis^39^. While thymopoiesis restores the T-cell repertoire at around 6 months upon auto-SCT, homeostatic proliferation is responsible for early reconstitution. However, it favors expansion of memory T-cells and reduces T-cell receptor diversity^40,41^. Fittingly, T-cells at early differentiation stages were less proliferative and less equipped with metabolic key proteins during engraftment. In addition, Ki-67 levels remained elevated for a longer period in CD4^+^ T-cells, which could be due to their protracted recovery. In this regard, CD8^+^ T-cells demonstrated a stronger upregulation of their glycolytic machinery, which could support their rapid reconstitution by glycolysis-derived carbon equivalents^42^.

CD56^bright^ NK-cells displayed a higher proliferation rate and enhanced metabolic parameters as compared to their CD56^dim^ counterparts. Both subsets are known to upregulate glycolysis and OPXHOS upon cytokine treatment with this effect being more pronounced in CD56^bright^ NK-cells^33^. These differences (together with our findings) could be explained by the increased expression of cytokine receptors on CD56^bright^ NK-cells^43^.

In contrast to T- and NK-cells, monocytes were not impacted quantitatively, which is in accordance with previous reports^44–46^. The monocyte composition was also not affected in contrast to findings that intermediate monocytes are less susceptible towards HDCT in MM patients^44^. IL-6 production was also preserved at all tested time points as a further indication of robustness of monocytes. Like T- and NK-cells, monocytes upregulated their metabolic machinery following auto-SCT, which is an established prerequisite for their expansion and funcionality^18,47^. Overall, proliferative activity and endowment with key metabolic molecules was stronger in intermediate monocytes upon auto-SCT, which is in opposition to findings during steady state conditions. Here, classical monocytes display the highest expression of glycolytic and non-classical monocytes of OXPHOS-related genes^48^. Interestingly, intermediate monocytes are known to expand during pro-inflammatory conditions like graft-versus-host disease^49,50^, rheumatoid arthritis^51^, and sepsis^52^, which could explain their increased proliferation and HK2 upregulation during engraftment. However, despite increased proliferation rates, we did not observe a significant proportional increase. This might be due to an increased susceptibility of CD16^+^ monocytes (including intermediate monocytes) towards spontaneous and ROS-induced apoptosis^53^, since we observed increased ROS levels in bulk monocytes during engraftment. Moreover, classical monocytes are known to upregulate CD16 on their surface under inflammatory conditions^54^, so the intermediate monocyte subset might be contaminated with activated classical monocyte leading to the observed increased proliferation and HK2 expression levels.

Finally, we observed that r/r lymphoma patients had persistently enhanced metabolic machinery in T-cells, NK-cells, and monocytes during first restaging.

Residual malignant cells below or above the conventional detection limit and/or defective cell intrinsic mechanisms that allow the activation to subside might cause these phenomena. In this context, at least two of the r/r lymphoma patients have already relapsed during our last analysis. In fact, chronic stimulation is known to cause exhaustion and senescence in immune cells, which results in inferior tumor control^55,56^. Within a mouse model of chronic lymphocytic choriomeningitis virus (LCMV) infection exhausted Eomes^high^ CD8^+^ T-cells had higher expression levels of Ki-67 compared to non-exhausted T-bet^high^ CD8^+^ T-cells. However, when transferred into infection-matched mice Eomes^high^ CD8^+^ T-cells proliferated less than T-bet^high^ ones^57^. Similar results were obtained when analyzing CD8^+^ tumor-infiltrating lymphocytes (TILs) exhibiting high expression levels of Ki-67 in terminal exhausted CD8^+^ T-cells^58^. In addition, microarray analysis of early exhausted T-cells within the LCMV mouse model demonstrated increased CPT1α and HIF1α gene expression levels in exhausted T-cells compared to memory ones early during infection. However, these differences were lost during infection persistence^59^.

Therefore, it would be of interest to study expression of immune checkpoint receptors like PD1, TIGIT or NKG2A, exhaustion markers, and the functionality of lymphocytes and monocytes in patients with r/r lymphoma in comparison to patients in remission.

Notably, increased proliferation of T_REG_ at day 28 upon auto-SCT is associated with a poor 5-year PFS in lymphoma patients^60^. We observed increased T_REG_ frequencies during engraftment with a consecutive decrease until the first restaging. We cannot rule out that the persistently increased metabolic T-cell activity in r/r lymphoma patient is due to proliferating T_REG_. However CD4^+^ T-cell counts decreased upon auto-SCT and their Ki-67 expression levels did not significantly differ between r/r lymphoma patients and those in remission in contrast to Ki-67 expression levels in CD8^+^ T-cells.

Strikingly, ALC did not differ in both groups, although lower ALC upon auto-SCT has been associated with reduced PFS and OS in lymphoma patients^10^.

Taken together, we characterized the metabolic profile of reconstituting immune cells following HDCT/auto-SCT for the first time. We were able to show that a persistently upregulated metabolic machinery in immune cells correlates with reduced PFS. However, the number of analyzed lymphoma patients in our study was too low (due to limited patient material and cell numbers) to appropriately establish a predictive pattern of the immune cell profile for PFS. Therefore, this data will require to be evaluated within a greater validation cohort and additional investigations are needed for understanding the underlying pathophysiology and to develop novel means of therapeutic interventions.

## Methods

### Study design and Patients’ characteristics

Healthy donors and patients with MM or lymphoma who underwent HDCT/auto-SCT between 2018 and 2020 were enrolled upon informed written consent in accordance with the Declaration of Helsinki. This study was approved by the local ethics committee of the University of Erlangen (no.: 313_17B and 200_12). We analyzed 53 patients (35 male, 18 female) with a median age of 60 years (range: 32–76 years). 30 patients suffered from MM and 23 from lymphoma including DLBCL (n = 13), TCL (n = 5), MCL (n = 2), Hodgkin’s Disease (HD) (n = 1), marginal zone lymphoma (MZoL) (n = 1), and follicular lymphoma (FL) (n = 1). Unfortunately, due to limited patient material and cell numbers we were not able to perform all of the listed experiments within all included patients. Blood from healthy donors was obtained from the blood bank of the Department of Transfusion Medicine and Haemostaseology of the University Hospital in Erlangen.

Peripheral blood (PB) was drawn at three time points: time point 1 (pre-HDCT) was before HDCT and at least two weeks after the start of the last therapy, time point 2 was during engraftment defined as the first (± 1) day after leukocyte regeneration (> 1000 leukocytes/µl), time point 3 was at restaging (post-SCT) (Supplementary Figure S14 online). Time between last conventional treatment and pre-HDCT time point was significantly longer in MM as compared to lymphoma patients (41 vs. 25 days; p < 0.0001). Time to engraftment was similar while the average time to restaging was different between myeloma and lymphoma patients based on our institutional guidelines. As anticipated the number of previous lines of therapy differs between diagnoses. The patients’ characteristics are summarized in Table 1.

### Blood samples, absolute cell numbers, and PBMC preparation

Absolute cell numbers were determined in whole blood immediately after collection via bead-based normalized flow cytometry (FACS) technology using Trucount Absolute Counting Tubes (Beckton Dickinson/BD Bioscience, New Jersey, USA). Prior to FACS analysis erythrocytes were lysed with BD FACS lysing solution. Reference values for absolute T-, NK-cell and monocyte counts within the blood of healthy donors were taken from the publication A.H. Kverneland et al. (cohort 1)^61^.

Peripheral blood mononuclear cells (PBMCs) were isolated from peripheral blood or leukocyte concentrates by density gravity centrifugation with Ficoll (General Electric/GE healthcare, Chicago, Illinois, USA) or Pancoll (PAN-Biotech, Aidenbach, Germany) using Leucosep tubes (Greiner Bio-One GmbH, Kremsmünster, Austria).

### Flow cytometry

Cell viability was assessed using the zombie aqua fixable viability dye (Biolegend, San Diego, California, USA). PBMCs were than either barcoded (procedure described later) or immediately stained following the manufacturers’ recommendations. For intracellular staining cells were fixed with BD Cytofix solution and washed in BD Perm/Wash solution (BD Bioscience).

The dyes 6-NBDG, Bodipy FL_C16_, CellROX Deep Red Reagent, MitoSox Red Mitochondrial Superoxide Indicator, MitoTracker Green FM (ThermoFisher Scientific), and TMRE (Cayman Chemical, Ann Arbor, Michigan) were used according to manufacturers’ instructions.

Samples were measured with a BD FACSCanto II or BD LSR Fortessa II flow cytometer using the BD FACSDiva Software. Data was analysed with the FlowJo V10 software (BD Bioscience).

A list of all antibodies and dyes can be found in Supplementary Table S4 online.

### Fluorescent cell barcoding technique

For optimizing comparability between different patient samples and to minimize interfering factors, fluorescent cell barcoding technique was used to stain PBMCs from all three time points of one donor together with an additional healthy donor (for standardization) in one well^62^. Briefly, cells were fixed with 2% paraformaldehyde (PFA) or BD Cytofix. Cells were then either carefully re-suspended in ice-cold 50% methanol (diluted in 0,9% NaCl) and incubated over night at -20 °C or washed twice with BD Perm/Wash solution (the latter method was needed in case surface epitopes of interest were denaturated by methanol). Subsequently, cells were labeled using different dilutions of the dye pacific blue (ThermoFisher Scientific). After three additional washing steps, all cells were re-suspended into one well and stained for additional surface or intracellular markers. Barcoding was deconvoluted by gating on the corresponding fluorescent cell barcoding dilution out of the whole cell population (Supplementary Figure S1 online).

### Intracellular cytokine detection

For intracellular cytokine staining cells were incubated for 6 h in complete media in a 96-well U-bottom plate with either 250 ng/ml Phorbol-12-myristat-13-acetat (PMA) and 1000 ng/mL Ionomycin or 1 µg/mL lipopolysaccharides (LPS). After the first two hours Brefeldin A was added, cells were harvested, labeled with fluorescent cell barcoding, and stained with antibodies against intracellular cytokines.

### Statistics

GraphPad Prism 6 or later versions (GraphPad, California, USA) and IBM SPSS Statistics 26 (IBM, New York, USA) were used for statistical analyses. Categorial variables were compared by χ^2^ test or Fisher´s exact test. Statistical significance was determined using either a Mann–Whitney test for unpaired, non-parametric variables or a Wilcoxon test for paired, non-parametric variables. A Chi-square test was used for categorical variables. For comparisons across time points of one variable, a Friedmann test with Dunn’s multiple comparison tests for repeated measurements was used. A two-way ANOVA with Sidak’s multiple comparison tests was used for paired comparisons between T- and NK-cell subsets and a Tukey’s multiple comparison tests for monocyte subsets across time points. For comparison of survival curves a Log-rank (Mantel Cox) test was performed. A p-value less than 0.05 was regarded statistically significant, statistical significance is indicated by the p-values (*p < 0.05; **p < 0.01; ***p < 0.001; ****p < 0.0001).

## Supplementary Information

Supplementary Information.

## Data Availability

The datasets generated during and/ or analyzed during the current study are available from the corresponding author on reasonable request.
