# Supplementary material for: The metabolic profile of reconstituting T-cells, NK-cells, and monocytes following autologous stem cell transplantation and its impact on outcome
Source: Sci Rep. 2022 Jul 6;12:11406. doi: 10.1038/s41598-022-15136-3 (PMC9259617; doi:10.1038/s41598-022-15136-3)
Supplement: Supplementary file 1 — Supplementary Information. [file 41598_2022_15136_MOESM1_ESM.docx]

**Supplementary information**

**The metabolic profile of reconstituting T-cells, NK-cells, and monocytes following autologous stem cell transplantation and its impact on outcome**

Silja Richter^1^, Martin Böttcher^1^, Simon Völkl^1^, Andreas Mackensen^1,5^, Evelyn Ullrich^2,3,4^, Benedikt Jacobs^1#^, Dimitrios Mougiakakos^1,5,6#^*

^1^Department of Internal Medicine 5, Hematology and Clinical Oncology, Friedrich-Alexander-Universität Erlangen-Nürnberg (FAU), University Hospital Erlangen, Erlangen, Germany

^2^Children’s Hospital, Goethe-University Frankfurt, Frankfurt, Germany

^3^Experimental Immunology, Goethe University Frankfurt, Frankfurt, Germany

^4^Frankfurt Cancer Institute, Goethe University, Frankfurt, Germany

^5^Deutsches Zentrum für Immuntherapie, Friedrich-Alexander-Universität Erlangen-Nürnberg (FAU), Erlangen, Germany

^6^Department of Hematology, Oncology, and Stem Cell Transplantation, Otto-von-Guericke-University Hospital Magdeburg, Magdeburg, Germany.

^#^These authors contributed equally to the work

*Corresponding author: Dimitrios Mougiakakos, Department of Hematology, Oncology, and Stem Cell Transplantation, Otto-von-Guericke-University Hospital Magdeburg, Leipziger Straße 44, 39120 Magdeburg, Germany. dimitrios.mougiakakos@med.ovgu.de. Phone: 0049 (0) 391/67-13266. Fax: -13267.

**Supplementary Table S1: Antibody combinations for the determination of T- and NK-cell as well as monocyte subsets**

| T-cells |
| --- |

| CD4^+^ T-cells | CD14^-^CD56^-^CD3^+^ CD4^+^ |
| --- | --- |
| CD8^+^ T-cells | CD14^-^CD56^-^CD3^+^ CD8^+^ |
| N | CD4^+^/CD8^+^ CD25^-^ CD127^+^ CD45RA^+^ CCR7^+^ |
| SCM | CD4^+^/CD8^+^ CD25^-^ CD127^+^ CD45RA^+^ CCR7^+^ CXCR3^+^ |
| RTE | CD4^+^ CD25^-^ CD127^+^ CD45RA^+^ CCR7^+^ CD31^+^ |
| CM | CD4^+^/CD8^+^ CD25^-^ CD127^+^ CD45RA^-^ CCR7^+^ |
| TM | CD4^+^/CD8^+^ CD25- CD127+ CD45RA- CCR7- CD28- |
| EM | CD4^+^/CD8^+^ CD25- CD127+ CD45RA- CCR7- CD28^+^ |
| EMRA | CD4^+^/CD8^+^ CD25- CD127+ CD45RA+ CCR7- CD28^-^ CD127^-^ |
| REG | CD4^+^ CD25^+^ CD127^-^ |

| NK-cells |
| --- |

| CD56^bright^ | CD14^-^CD3^-^CD56^++^CD16^-^ |
| --- | --- |
| CD56^dim^ | CD14^-^CD3^-^CD56^+^CD16^-/+^ |

| Monocytes |
| --- |

| Classical | CD3^-^CD56^-^ CD14^++^CD16^-^ |
| --- | --- |
| Intermediate | CD3^-^CD56^-^ CD14^+^CD16^+^ |
| Non-classical | CD3^-^CD56^-^ CD14^dim^CD16^+^ |

N, naïve; SCM, stem-cell memory; RTE, recent thymic emigrants; CM, central memory; TM, transitional memory; EM, effector memory; EMRA, effector memory re-expressing CD45RA; REG, regulatory T-cells.

**Supplementary Table S2: Distribution of the various differentiation stages of CD4^+^ and CD8^+^ T-cells during HDCT/ auto-SCT**

Distribution of various differentiation stages of CD4^+^ and CD8^+^ T-cells (n: 10) including naïve (N), recent thymic emigrants (RTE), stem cell memory (SCM), central memory (CM), transitional memory (TM), effector memory (EM), effector memory CD45RA^+^ (EMRA) and regulatory T-cells (REG)*.* All results are shown as median values of percentages of parent (CD4^+^or CD8^+^ T-cells). Statistical significance was determined using a Friedman test with Dunn’s multiple comparison tests for repeated measurements (n:10). *P < .05; **P < .01; ***P < .001.

**Supplementary Table S3: Patient characteristics of progressive vs. non-progressive lymphoma patients within the first year upon auto-SCT**

|  |  | **Refractory/Relapsed** | **Remission** |
| --- | --- | --- | --- |
| **Number of patients** |  | 7 | 14 |
|  | DLBCL | 4 | 8 |
|  | MCL |  | 2 |
|  | MZoL |  | 1 |
|  | FL |  | 1 |
|  | HD |  | 1 |
|  | TCL | 3 | 1 |
| **Gender** | male | 5 | 10 |
|  | female | 2 | 4 |
| **Age, median (range)** |  | 59 (51-74) | 59 (32-76) |
| **Therapy line, median (range)** |  | 2 (1-2) | 2 (1-3) |
| **Time from last chemo to start of HDT, median (range)** |  | 21 (17-42) | 27.5 (20-40) |
| **Time from SCT to engraftment, median (range)** |  | 12 (10-14) | 10.5 (9-13) |
| **Time from SCT to re-staging, median (range)** |  | 33  (23-45) | 34.5  (30-57) |
| **Time from restaging to detection of relapse, median (range)** |  | 49  (-18-99) | - |
| **CD34^+^ cell number (x10^6^/ kg), median (range)** |  | 4.14  (3.1-5.7) | 4.51  (2.6-17.6) |
| **ALC pre-HDCT (count/µl), median (range)** |  | 1058.0 (197.5-2686.6) | 967.3 (383.8-2356) |
| **ALC engraftment (count/µl), median (range)** |  | 203.4 (47.6-1978.2) | 163.8 (12.4-1060.2) |
| **ALC post-SCT (count/µl), median (range)** |  | 1311.2 (317.0-5097.2) | 1393.4 (280.3-2369.4) |

DLBCL, diffuse large B-cell lymphoma; MCL, mantle cell lymphoma; MZol, marginal zone lymphoma; FL, follicular lymphoma; HD, Hodgkin’s disease; TCL, T-cell lymphoma; ALC, absolute lymphocyte count.

Statistical significance was determined using a Mann-Whitney test for unpaired, non-parametric variables or a Chi-square test for categorical variables. P values comparing refractory/ relapsing and non-relapsing lymphoma patients within 1 year upon auto-SCT.

**Supplementary Table S4: Antibodies and dyes**

| **Monoclonal antibody/ dye** | **Fluoro-chrome** | **Clone** | **Use** | **Vendor** | **Order number** | **Dilution** |
| --- | --- | --- | --- | --- | --- | --- |
| **6-NBDG**  (6-(N-(7-Nitrobenz-2-oxa-1,3-diazol-4-yl)amino)-6-desoxyglucose) | |  | Glucose uptake | ThermoFisher Scientific | N23106 | 1,5:200 |
| **BODIPY FL_C16_** (4,4-Difluoro-5,7-Dimethyl-4-Bora-3a,4a-Diaza-s-Indacene-3-Hexadecanoic Acid) | |  | Fatty acid uptake | ThermoFisher Scientific | D-3821 | 2,5µM |
| **CD003** | PE/Cyanine7 | SK2 |  | BioLegend | 344816 | 01:20 |
| **CD003** | BUV737 | UCHT-1 | various | BD Bioscience | 612750 | 01:20 |
| **CD003** | PeCy7 | SK7 | various | BioLegend | 344816 | 01:50 |
| **CD004** | BUV395 | SK3 | various | BD Bioscience | 563550 | 01:20 |
| **CD008** | BUV496 | RPA-T8 | various | BD Bioscience | 612942 | 01:20 |
| **CD014** | Per-CP Cy5.5 | HCD14 | various | BioLegend | 325622 | 01:10 |
| **CD014** | APC | HCD14 | Trucount | BioLegend | 325608 | 01:50 |
| **CD014** | BV510 | M5E2 | Cytokine secretion | BioLegend | 301842 | 01:50 |
| **CD016** | APC/Cyanine7 | 3G8 | various | BioLegend | 392918 | 01:25 |
| **CD016** | FITC | 3G8 | various | BioLegend | 302006 | 01:20 |
| **CD019** | BV510 | HIB19 | various | BioLegend | 302242 | 01:20 |
| **CD025** | PE/Dazzle 594 | BC96 | T cell subsets | BioLegend | 302646 | 01:50 |
| **CD028** | PerCP Cy5.5 | CD28.2 | T cell subsets | BioLegend | 302922 | 01:10 |
| **CD031** | APC/Cy7 |  | T cell subsets | BioLegend | 303120 | 01:10 |
| **CD045** | PE | HI30 | Trucount | BioLegend | 304039 | 01:50 |
| **CD045** | PE/Cyanine7 | 2D1 | various | BioLegend | 368532 | 01:50 |
| **CD045RA** | BV785 | H100 | T cell subsets | BioLegend | 304140 | 01:10 |
| **CD056** | ECD Texas Red | N901 (NKH-1) | various | Beckman Coulter | A82943 | 01:10 |
| **CD056** | APC Cy7 | HCD56 | various | BioLegend | 318332 | 01:10 |
| **CD056** | BV421 | HCD56 | various | BioLegend | 318328 | 01:50 |
| **CD127 (IL-7Rα)** | BV711 | A019D5 | T cell subsets | BioLegend | 351328 | 01:10 |
| **CD183 (CXCR3)** | BV605 | G025H7 | T cell subsets | BioLegend | 353728 | 01:10 |
| **CD197 (CCR7)** | PE/Cyanine7 | G043H7 | T cell subsets | BioLegend | 353226 | 01:10 |
| **CellROX Deep Red Reagent** | |  | Detection of cellular ROS levels | ThermoFisher Scientific | C10422 | 5µM |
| **CPT1A** | AF 488 | 8F6AE9 | Barcoding metabolism Panel and T/ NK cell subsets | Abcam | ab171449 | 01:50 |
| **FoxP3** | FITC |  | T cell subsets | BioLegend | 320106 | 01:12,5 |
| **GLUT1** | APC | FAB1418A | Barcoding metabolism Panel | R&D Systems | 202915 | 01:10 |
| **Hexokinase II** | - | EPR20839 | Barcoding metabolism Panel | Abcam | ab209847 | 01:200 |
| **IFN-g** | APC-Vio770 | Clone REA600 | Cytokine production | Miltenyi biotec | 130-114-022 | 01:50 |
| **IL-2** | FITC | Clone REA689 | Cytokine production | Miltenyi biotec | 130-111-488 | 01:50 |
| **IL-4** | PE | Clone REA895 | Cytokine production | Miltenyi biotec | 130-114-884 | 01:50 |
| **IL-6** | FITC | REA1037 | Cytokine production | Miltenyi biotec | 130-117-589 | 01:50 |
| **Ki-67** | FITC | 20Raj1 | Barcoding metabolism Panel | eBioscience™ ThermoFisher Scientific | 11-5699-42 | 01:10 |
| **Ki-67** | PE | Ki67 | T / NK cell subsets | BioLegend | 350504 | 01:10 |
| **MitoSox Red Mitochondrial Superoxide Indicator** | |  | Detection of mitochondrial ROS | ThermoFisher Scientific | M36008 | 5µM |
| **MitoTracker Green FM** | |  | Detection of mitochondrial mass | ThermoFisher Scientific | M7514 | 25nM |
| **Pacific Blue** | BV421 |  | Barcoding | ThermoFisher Scientific | P10163 | 3,78  µg/ml |
| **Tetramethylrhodamine, Ethyl Ester, Perchlorate (TMRE)** | |  | Mitochondrial membrane detection | Cayman Chemical | 21426 | 100nM |
| **TNF** | APC | REA656 | Cytokine production | Miltenyi biotec | 130-120-149 | 01:50 |
| **TNF** | PeVio770 | cA2 | Cytokine production | Miltenyi biotec | 130-120-630 | 01:50 |
| **Zombie Aqua Fixable Viability DCM** | BV510 |  | Live-dead marker | BioLegend | 423102 | 01:100 |
| **Goat Anti-Rabbit IgG sek. AK** | AF647 |  | Secondary antibody to HK2 | Abcam | ab150079 | 01:50 |

**Supplementary Figure S1**

**Supplementary Figure S1: Gating strategies for the analysis of absolute immune cell counts and deconvolusion of barcoded cells**

(A) Representative flow cytometry plots with consecutive gates are shown for the analysis of absolute cell counts of T- and NK-cells as well as monocytes using Trucount Absolute Counting Tubes (BD). In addition, the gating strategies for the deconvolusion of barcoded (B) T- and NK-cells as well as (C) monocytes are illustrated. (D) Finally, representative flow cytometry plots with consecutive gates to identify CD4^+^ and CD8^+^ differentiation subsets are presented.

**Supplementary Figure S2**

**Supplementary Figure 2: Absolute cells counts during the restaging appointment did not differ between myeloma and lymphoma patients**

Analysis of absolute (**A**) CD3^+^ T-cell, (**B**) NK-cell, and (**C**) monocyte count in MM (n:18-19) and lymphoma (n: 17-19) patients (mean +/- standard deviation SD) during HDCT/ auto-SCT. The mean of a healthy donor control group (n: 52 from A.H. Kverneland et al.) is shown as a horizontal dashed line for reference. Statistical significance was determined using a Mixed-model effect analysis (two-way ANOVA) with Sidak’s multiple comparison tests.

**Supplementary Figure S3**

**Supplementary Figure S3: Mitochondrial ROS production is significantly increased only in NK-cells, but not in T-cells or monocytes upon HDCT/auto-SCT**

Mitochondrial ROS production in (A) T-cells, (B) NK-cells and (C) monocytes was measured using MitoSox. All results were plotted as median fluorescence intensity (MdFI) for all cell populations (n: 15, mean values +/- SD). The mean of a healthy donor control group (n: 10) is shown as a horizontal dashed line for reference. Statistical significance was determined using a Friedman test with Dunn’s multiple comparison tests for repeated measurements. *P < .05; ***P < .001.

**Supplementary Figure S4**

**Supplementary Figure S4: ROC-Analysis for determination of an optimal cut-off value for HK2 expression in NK-cells post-SCT between lymphoma patients in remission vs. refractory/ relapsing (r/r) patients**

(A) HK2 expression levels in NK-cells post-SCT were plotted for lymphoma patients in remission vs. r/r ones. (B) A ROC analysis of the HK2 expression in NK-cells post-SCT was performed. (C) Data table shows sensitivity (%) and specificity (%), as well as the 95% confidence interval and Likelihood ratio (LR) for different HK2 (MdFI) expression cutoff values. Cut off value was selected according to highest sensitivity, specificity, and high LR (circle).

**Supplementary Figure S5**

**Supplementary Figure S5: ROC-Analysis for determination of an optimal cut-off value for Ki-67^+^ T-cells post-SCT between lymphoma patients in remission vs. refractory/ relapsing (r/r) patients**

(A) Ki-67 expression levels in T-cells post-SCT were plotted for lymphoma patients in remission vs. r/r ones. (B) A ROC analysis of the Ki-67 expression in T-cells post-SCT was performed. (C) Data table shows sensitivity (%) and specificity (%), as well as the 95% confidence interval and Likelihood ratio (LR) for different Ki-67 (%) expression cutoff values. Cut off value was selected according to highest sensitivity, specificity, and high LR (circle).

**Supplementary Figure S6**

**Supplementary Figure S6: ROC-Analysis for determination of an optimal cut-off value for Ki-67^+^ NK-cells post-SCT between lymphoma patients in remission vs. refractory/ relapsing (r/r) patients**

(A) Ki-67 expression levels in NK-cells post-SCT were plotted for lymphoma patients in remission vs. r/r ones. (B) A ROC analysis of the Ki-67 expression in NK-cells post-SCT was performed. (C) Data table shows sensitivity (%) and specificity (%), as well as the 95% confidence interval and Likelihood ratio (LR) for different Ki-67 (%) expression cutoff values. Cut off value was selected according to highest sensitivity, specificity, and high LR (circle).

**Supplementary Figure S7**

**Supplementary Figure S7: ROC-Analysis for determination of an optimal cut-off value for CPT1α expression in T-cells post-SCT between lymphoma patients in remission vs. refractory/ relapsing (r/r) patients**

(A) CPT1α expression levels in T-cells post-SCT were plotted for lymphoma patients in remission vs. r/r ones. (B) A ROC analysis of the CPT1α expression in T-cells post-SCT was performed. (C) Data table shows sensitivity (%) and specificity (%), as well as the 95% confidence interval and Likelihood ratio (LR) for different CPT1α (MdFI) expression cutoff values. Cut off value was selected according to highest sensitivity, specificity, and high LR (circle).

**Supplementary Figure S8**

**Supplementary Figure S8: ROC-Analysis for determination of an optimal cut-off value for CPT1α expression in monocytes post-SCT between lymphoma patients in remission vs. refractory/ relapsing (r/r) patients**

(A) CPT1α expression levels in monocytes post-SCT were plotted for lymphoma patients in remission vs. r/r ones. (B) A ROC analysis of the CPT1α expression in monocytes post-SCT was performed. (C) Data table shows sensitivity (%) and specificity (%), as well as the 95% confidence interval and Likelihood ratio (LR) for different CPT1α (MdFI) expression cutoff values. Cut off value was selected according to highest sensitivity, specificity, and high LR (circle).

**Supplementary Figure S9**

**Supplementary Figure S9: ROC-Analysis for determination of an optimal cut-off value for CPT1α expression in NK-cells post-SCT between lymphoma patients in remission vs. refractory/ relapsing (r/r) patients**

(A) CPT1α expression levels in NK-cells post-SCT were plotted for lymphoma patients in remission vs. r/r ones. (B) A ROC analysis of the CPT1α expression in NK-cells post-SCT was performed. (C) Data table shows sensitivity (%) and specificity (%), as well as the 95% confidence interval and Likelihood ratio (LR) for different CPT1α (MdFI) expression cutoff values. Cut off value was selected according to highest sensitivity, specificity, and high LR (circle).

**Supplementary Figure S10**

**Supplementary Figure S10: Persistently increased proliferation in CD8^+^ as well as CPT1α expression in CD4^+^ and CD8^+^ T-cells upon HDCT/ auto-SCT is associated with refractory disease or early relapse in lymphoma**.

(**A**) Multiple unpaired Mann-Whitney tests for Ki-67, HK2 and CPT1α expression in CD4^+^ and CD8^+^ T-cells at the post-SCT time point were performed to identify significant differences between lymphoma patients being refractory or relapsed (r/r) within the first year upon SCT and lymphoma patients being still in remission (remission) at this time point. P-values were adjusted using False-Discovery-Rate adjustment. (**B-D**) Area under the Receiver operating characteristic (ROC) curve analysis was used to define an optimal cut-off value for each parameter for further evaluation and grouping for survival analysis (Supplementary Figure S14-16). Cut-off values were selected according to highest sensitivity (true positive rate) and specificity (true negative rate) and high Likelihood ratio (LR). Tumor progress within one year was recorded as an event. For comparison of progression free survival curves a Log-rank (Mantel Cox) test was performed (n: 7 refractory/ relapsing and 14 non-relapsing lymphoma patients). **P < .01.

**Supplementary Figure S11**

**Supplementary Figure S11: ROC-Analysis for determination of an optimal cut-off value for Ki-67 expression in CD8^+^ T-cells post-SCT between lymphoma patients in remission vs. refractory/ relapsing (r/r) patients**

(A) Ki-67 expression levels in CD8^+^ T-cells post-SCT were plotted for lymphoma patients in remission vs. r/r ones. (B) A ROC analysis of the Ki-67 expression in CD8^+^ T-cells post-SCT was performed. (C) Data table shows sensitivity (%) and specificity (%), as well as the 95% confidence interval and Likelihood ratio (LR) for different Ki-67 (%) expression cutoff values. Cut off value was selected according to highest sensitivity, specificity, and high LR (circle).

**Supplementary Figure S12**

**Supplementary Figure S12: ROC-Analysis for determination of an optimal cut-off value for CPT1α expression in CD4^+^ T-cells post-SCT between lymphoma patients in remission vs. refractory/ relapsing (r/r) patients**

(A) CPT1α expression levels in CD4^+^ T-cells post-SCT were plotted for lymphoma patients in remission vs. r/r ones. (B) A ROC analysis of the CPT1α expression in CD4^+^ T-cells post-SCT was performed. (C) Data table shows sensitivity (%) and specificity (%), as well as the 95% confidence interval and Likelihood ratio (LR) for different CPT1α (MdFI) expression cutoff values. Cut off value was selected according to highest sensitivity, specificity, and high LR (circle).

**Supplementary Figure S13**

**Supplementary Figure S13: ROC-Analysis for determination of an optimal cut-off value for CPT1α expression in CD8^+^ T-cells post-SCT between lymphoma patients in remission vs. refractory/ relapsing (r/r) patients**

(A) CPT1α expression levels in CD8^+^ T-cells post-SCT were plotted for lymphoma patients in remission vs. r/r ones. (B) A ROC analysis of the CPT1α expression in CD8^+^ T-cells post-SCT was performed. (C) Data table shows sensitivity (%) and specificity (%), as well as the 95% confidence interval and Likelihood ratio (LR) for different CPT1α (MdFI) expression cutoff values. Cut off value was selected according to highest sensitivity, specificity, and high LR (circle).

**Supplementary Figure S14**

**Supplementary Figure S14: Study design and disease distribution**

(A) The course of the absolute leukocyte count (10^3^/µl) of patients receiving HDCT with subsequent auto-SCT is indicated over several weeks. In the current study blood samples were taken at three different time points: before the start of HDCT (pre-HDCT), during engraftment defined as the first (+/-1) day after leukocyte regeneration (>1000 leukocytes/µl) and at restaging around 34-45 days after SCT (post-SCT).
